# [Retracted] MicroRNA‑329‑3p targets MAPK1 to suppress cell proliferation, migration and invasion in cervical cancer

**DOI:** 10.3892/or.2024.8745

**Published:** 2024-04-29

**Authors:** Wenfeng Li, Jingjing Liang, Zhechao Zhang, Hongyan Lou, Liang Zhao, Yunsheng Xu, Rongying Ou

Oncol Rep 37: 2743–2750, 2017; DOI: 10.3892/or.2017.5555

Following the publication of the above article, a concerned reader drew to the Editor's attention that certain of the Transwell cell migration and invasion assay data featured in Figs. 5C and 6C were strikingly similar to data appearing in different form in other articles written by different authors at different research institutes that had already been published elsewhere prior to the submission of this paper to *Oncology Reports*, or were submitted for consideration for publication at around the same time.

In view of the fact that certain of these data had already apparently been published prior to the submission of this article for publication, the Editor of *Oncology Reports* has decided that this paper should be retracted from the Journal. The authors were asked for an explanation to account for these concerns, but the Editorial Office did not receive a reply. The Editor apologizes to the readership for any inconvenience caused.

